# Comparative genomic analysis of the secondary flagellar (*flag*-2) system in the order Enterobacterales

**DOI:** 10.1186/s12864-020-6529-9

**Published:** 2020-01-30

**Authors:** Pieter De Maayer, Talia Pillay, Teresa A. Coutinho

**Affiliations:** 1 School of Molecular & Cell Biology, University of the Witwatersrand, 2050 Wits, Johannesburg, South Africa; 2 Centre for Microbial Ecology and Genomics, University of Pretoria 0002, Pretoria, South Africa

**Keywords:** Enterobacterales, *flag*-2, primary and secondary flagellar system, Flagellin glycosylation, Motility

## Abstract

**Background:**

The order Enterobacterales encompasses a broad range of metabolically and ecologically versatile bacterial taxa, most of which are motile by means of peritrichous flagella. Flagellar biosynthesis has been linked to a primary flagella locus, *flag*-1, encompassing ~ 50 genes. A discrete locus, *flag*-2, encoding a distinct flagellar system, has been observed in a limited number of enterobacterial taxa, but its function remains largely uncharacterized.

**Results:**

Comparative genomic analyses showed that orthologous *flag*-2 loci are present in 592/4028 taxa belonging to 5/8 and 31/76 families and genera, respectively, in the order Enterobacterales. Furthermore, the presence of only the outermost *flag-*2 genes in many taxa suggests that this locus was far more prevalent and has subsequently been lost through gene deletion events. The *flag*-2 loci range in size from ~ 3.4 to 81.1 kilobases and code for between five and 102 distinct proteins. The discrepancy in size and protein number can be attributed to the presence of cargo gene islands within the loci. Evolutionary analyses revealed a complex evolutionary history for the *flag*-2 loci, representing ancestral elements in some taxa, while showing evidence of recent horizontal acquisition in other enterobacteria.

**Conclusions:**

The *flag*-2 flagellar system is a fairly common, but highly variable feature among members of the Enterobacterales. Given the energetic burden of flagellar biosynthesis and functioning, the prevalence of a second flagellar system suggests it plays important biological roles in the enterobacteria and we postulate on its potential role as locomotory organ or as secretion system.

## Background

The order Enterobacterales encompasses a diverse group of Gram-negative, non-sporing, facultatively anaerobic rod-shaped bacteria. Recent phylogenomic re-evaluation of the sole family in this order, the *Enterobacteriaceae*, has resulted in its division into eight distinct families [1]. Members of this order can be found in a diverse range of environments including air, soil, water and in association with plant and animal hosts, and include some of the most important pathogens of these hosts [2]. Key to the ecological success of enterobacteria is their capacity for motility, which is largely mediated by flagella, specialized surface structures that allow bacterial cells to move along surfaces, towards nutrients and away from harmful substances [3]. Furthermore, flagella play crucial roles in enterobacterial pathogenesis, contributing to adherence, invasion and colonization of host cells and tissues [4, 5].

Flagella are highly complex structures, comprised of three major components, a basal body, hook and filament [6]. The basal body anchors the flagellum to the cell envelope and incorporates the flagellar motor [3, 6]. The flagellar hook connects the basal body to the flagellar filament and acts as a universal joint, facilitating dynamic and efficient motility and taxis [7, 8]. The filament is the longest, surface-exposed, component of the bacterial flagellum and is composed of approximately 20,000 subunits of the major structural protein [6, 9]. This filament serves as a propeller, which converts the motor into thrust to propel the bacterial cell [9]. Typically, up to 50 genes are required for the assembly, maintenance and functioning of these surface appendages [10]. In the model enterobacterial taxa *Escherichia coli* and *Salmonella enterica*, the genes involved in flagellar biosynthesis and functioning are located in three genomic clusters, collectively termed the primary flagellar locus (*flag*-1) [11, 12]. Although most of the genes involved in flagellar biosynthesis are common to most bacterial taxa, a high level of divergence in flagellar structure exists and allows different microorganisms to be distinguished from one another [10]. Furthermore, flagellin glycosylation and methylation has been observed in a number of bacterial species and has shown to play a crucial role in flagella assembly and virulence [13, 14].

In addition to the primary flagellar system, a number of enterobacterial taxa, namely *E. coli*, *Yersinia enterocolitica*, *Yersinia pestis* and *Citrobacter rodentium*, have been observed to possess a distinct secondary flagellar (*flag*-2) system [15, 16]. This *flag*-2 system has been attributed to a specific genomic locus, which resembles that coding for the lateral flagella in *Aeromonas hydrophila* and *Vibrio parahaemolyticus*, and is genetically distinct from the gene clusters that are required for the biosynthesis of the primary flagellar system [15]. The *flag-*2 locus of *E. coli* 042 is ~ 48.8 kb in size and codes for 44 distinct proteins involved in the synthesis. This second flagellar system has been suggested to facilitate swarming motility on solid surfaces [15]. Knock-out mutagenesis of the *Y. enterocolitica flag-*2 system, however, had no effect on motility, and it was suggested to serve as a virulence factor that aids this pathogen gain entry into mammalian cells [16]. Here, by means of comparative genomic analyses, we have further analysed the *flag*-2 locus and show it to present in a substantial number of taxa across a broad spectrum of the genera and families in the order Enterobacterales. The enterobacterial *flag*-2 locus comprises a large set of conserved genes for the synthesis and functioning of the secondary flagellar system, but also incorporates variable regions that may contribute to both structural and functional versatility of this system. Our genomic analysis suggests that the *flag-*2 locus may have been universally present in some enterobacterial lineages, and that this ancestral locus has subsequently been lost in some taxa, while in other lineages it has been derived through horizontal gene acquisition. Finally, we postulate on the potential functions of this versatile and widespread flagellar system in the Enterobacterales.

## Results and discussion

### The *flag*-2 locus is widespread among the Enterobacterales

**Fig. 1:**
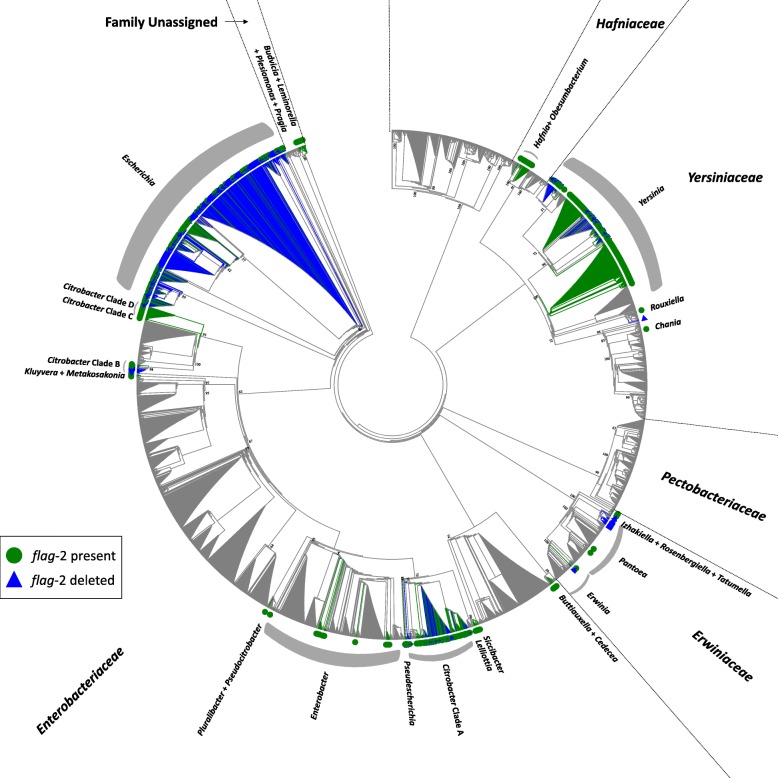
Distribution of the *flag*-2 locus across the order Enterobacterales. A circularized, topology-only ML phylogeny was constructed on the basis of the concatenated alignments of the house-keeping proteins GyrB, InfB, RecA and RpoB. The tree was constructed on a trimmed concatenated alignment of 2613 amino acid sites and using the best-fit evolutionary model JTTDCMut+I + G4. Bootstrap values (*n* = 1000 replicates) > 50% for the major clades are shown. Strains whose genome incorporates the *flag*-2 locus are indicated by green dots, while those where deletion between *lfhA* and *lafU* has occurred are indicated by blue triangles

The finished and draft genomes of 4028 bacterial strains encompassing the taxonomic diversity of the order Enterobacterales were screened for the presence of *flag-*2 loci (Additional file 1: Table S1). A total of 592 (15% of the analysed taxa) strains were observed to possess an orthologous locus (Fig. 1 – indicated by green circles; Additional file 1: Table S1) and these are distributed across a wide taxonomic breadth of the order. As such, *flag*-2 loci occur in five of the eight families and 31/76 genera included in this study. Exceptions are observed for the families *Morganellaceae* (7 genera – 313 strains), *Pectobacteriaceae* (7 genera – 244 strains) and *Thorselliaceae* (*Thorsellia* – 1 strain), where no *flag*-2 loci occur. The highest prevalence can be observed in the family *Budvicaceae* (7/9 studied taxa) and *Yersiniaceae* (225/605 strains), while only 13% (316/2464 taxa) of the family *Enterobacteriaceae* contained orthologous loci (Fig. 1; Table 1). Differences in prevalence at the genus level could also be observed. Notably, *flag*-2 loci are universally present in several genera, including *Citrobacter* Clade D (30/30 strains) and *Plesiomonas* (8/8 strains), while in the two genera with the highest number of *flag*-2 loci present, *Yersinia* (222/394 strains) and *Escherichia* (124/522 strains), 56 and 24% of the evaluated strains encode *flag*-2 systems, respectively. In some genera, the presence of *flag*-2 loci represents a rare trait. For example, only two of 151 analysed *Pantoea* strains contain *flag*-2 loci. Diversity in terms of *flag*-2 locus presence can furthermore be observed at the species level. For example, all 100 of the evaluated *Y. pestis* strains incorporate a *flag*-2 locus, while it only occurs in 24/100 *Escherichia coli* strains.

**Table 1:** Proportion of Enterobacterales families and genera where *flag*-2 loci are present

	*#* strains	% containing *flag*-2 locus	# genera/species	% Genera/species with *flag*-2 loci
***Budviciaceae***	**9**	**78%**	**4**	**75%**
*Budvicia*	2	100%	1	100%
*Leminorella*	2	50%	2	50%
*Limnobaculum*	1	–	1	–
*Pragia*	4	100%	1	100%
***Enterobacteriaceae***	**2464**	**13%**	**32**	**53.13%**
*Atlantibacter*	5	–	2	–
*Buttiauxella*	8	38%	7	28.57%
*Cedeceae*	8	75%	7	71.42%
*Citrobacter* Clade A	157	58%	10	80.00%
*Citrobacter* Clade B	21	24%	4	75.00%
*Citrobacter* Clade C	4	100%	1	100.00%
*Citrobacter* Clade D	30	100%	1	100.00%
*Cronobacter*	189	–	7	–
*Enterobacter*	608	5%	28	21.43%
*Escherichia*	522	24%	8	87.50%
*Franconibacter*	10	–	2	–
*Klebsiella* Clade A	100	–	1	–
*Klebsiella* Clade B	310	–	4	–
*Klebsiella* Clade C	189	–	3	–
*Kluyvera*	9	44%	6	66.67%
*Kosakonia*	24	–	9	–
*Leclercia*	10	–	2	–
*Lelliottia*	13	69%	6	66.67%
*Mangrovibacter*	2	100%	2	100.00%
*Metakosakonia*	3	33%	1	33.33%
*Phytobacter*	2	–	2	–
*Pluralibacter*	17	12%	1	100.00%
*Pseudescherichia*	2	100%	1	100.00%
*Pseudocitrobacter*	1	100%	1	100.00%
*Raoultella*	85	–	4	–
*Salmonella*	112	–	2	–
*Shimwellia*	2	–	1	–
*Siccibacter*	6	33%	2	50%
*Superficieibacter*	2	–	1	–
*Trabulsiella*	8	–	3	–
*Yokenella*	5	–	1	–
***Erwiniaceae***	**287**	**2%**	**8**	**50%**
*Buchnera*	58	–	42	–
*Erwinia*	63	3%	17	12%
*Izhakiella*	2	100%	2	100%
*Mixta*	4	–	4	–
*Pantoea*	151	1%	25	8%
*Phaseolibacter*	1	–	1	–
*Rosenbergiella*	1	100%	1	100.00%
*Tatumella*	5	20%	4	25%
*Wigglesworthia*	2	–	2	–
***Hafniaceae***	**97**	29%	4	50%
*Edwarsiella*	50	–	5	–
*Enterobacillus*	1	–	1	–
*Hafnia*	42	57%	3	100%
*Obesumbacterium*	4	100%	1	100%
***Morganellaceae***	**313**	**0%**	**7**	**0%**
*Arsenophonus*	2	–	1	–
*Moellerella*	2	–	1	–
*Morganella*	55	–	5	–
*Photorhabdus*	31	–	9	–
*Proteus*	122	–	14	–
*Providencia*	58	–	14	–
*Xenorhabdus*	43	–	24	–
***Pectobacteriaceae***	**244**	**–**	**7**	**–**
*Biostraticola*	1	–	1	–
*Brenneria*	9	–	6	–
*Dickeya*	55	–	9	–
*Lonsdalea*	35	–	4	–
*Pectobacterium*	140	–	16	–
*Samsonia*	1	–	1	–
*Sodalis*	3	–	3	–
***Thorselliaceae***	**1**	–	**1**	**0%**
*Thorsellia*	1	–	1	–
***Yersiniaceae***	**605**	**37%**	**12**	**25.00%**
*Chania*	2	50%	2	50%
*Ewingella*	2	–	1	–
*Gibsiella*	4	–	1	–
*Nissabacter*	1	–	1	–
*Rahnella*	18	–	7	–
*Rouxiella*	3	33%	3	33.33%
*Serratia* Clade A	159	–	10	–
*Serratia* Clade B	12	–	1	–
*Serratia* Clade C	8	–	2	–
*Serratia* Clade D	1	–	1	–
*Serratia* Clade E	1	–	1	–
*Yersinia*	394	56%	24	62.50%
**Family Unassigned**	**8**	**100%**	**1**	**100%**
*Plesiomonas*	8	100%	1	100%
Overall	4028	15%	72 genera	43.06%

The families in the order Enterobacterales incorporated in this study, and the prevalence of *flag*-2 loci among them are indicated in bold

### Molecular architecture of the *flag-*2 loci

**Fig. 2:**
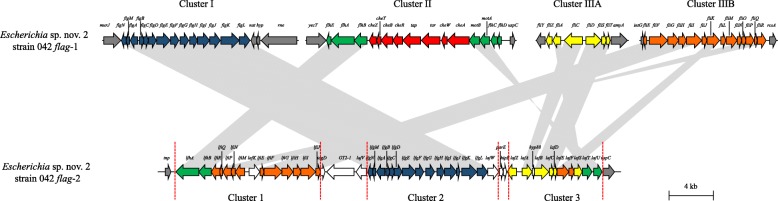
Schematic comparison of the *flag*-1 and *flag*-2 loci of *Escherichia* sp. nov. 2 strain 042. The *flag*-2 genes are coloured in accordance with orthology to conserved genes in the *flag*-1 locus. A scale bar (4 kilobases) indicates the size of the loci

**Fig. 3:**
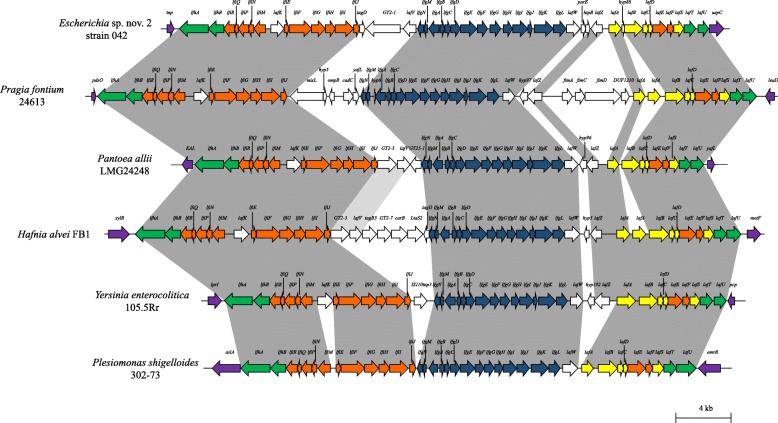
Schematic comparison of the *flag*-2 loci of representatives of each family within the order Enterobacterales. Flanking genes are coloured in purple, while the *flag*-2 loci core genes are coloured in accordance with orthology to conserved genes in the *flag*-1 locus (Fig. 2). Dark grey shading indicates orthology between core *flag*-2 genes, while the light grey shading indicates conservation of genes in the variable regions. A scale bar (4 kilobases) indicates the size of the loci

The *flag*-2 loci comprise of a set of co-localised genes within the genomes of the enterobacteria that harbour them (Fig. 2). This is in contrast to the *flag*-1 system (Fig. 3), where gene loci responsible for the synthesis and functioning of the primary flagellar system are generally dispersed across the enterobacterial chromosome [12]. The enterobacterial *flag-*2 loci range in size from ~ 3.4 to ~ 81.8 kilobases (average 38.1 kb) and code for between five and 102 (average 43 proteins) proteins (Additional file 1: Table S2). The discrepancy in size and number of proteins encoded by the loci can largely be attributed to frequent deletions and insertion of non-core genes within the loci. Substantially larger *flag*-2 loci are observed in *Escherichia albertii* B156, *Citrobacter* (Clade A) sp. nov 1 S1285 and three *C. rodentium* strains. This can be linked to the insertion of prophage elements within the *flag*-2 loci, contributing on average 37.7 kb of sequence and 54 proteins.

Comparative analysis showed extensive synteny and sequence conservation among the *flag-*2 loci (Fig. 2). Of the 592 strains with *flag*-2 loci, 461 (77.87% of strains with *flag*-2 loci) encode an orthologue complement of 39 conserved proteins. One of these conserved proteins is LafA, the flagellin counterpart of the *flag*-2 system, which is present in multiple copies in 156/592 (26.35%) strains, with up to five copies (*Y. pestis* Pestoides B – 81.81% average amino acid identity) encoded by the *flag*-2 locus. Multiple copies of the flagellin gene have also been observed in the *flag*-1 loci of many enterobacteria and have been suggested to contribute to the phenomenon of phase variation [14, 17, 18]. As flagellin proteins are potent antigens, the phase variable expression of these proteins may enable these organisms to temporarily avoid immune responses in both plant and animal hosts [14, 18]. The remaining 38 single-copy orthologues share an average amino acid identity (AAI) of 61.13% among the 461 enterobacteria with complete *flag*-2 loci.

In accordance with the study on the *flag*-2 locus of *Escherichia* sp. nov. 2 strain 042, the *flag*-2 loci can be subdivided into three distinct gene clusters – Cluster 1–3 (Fig. 2) [15]. Cluster 1, comprised of fourteen genes *lfhAB-lfiRQPNM-lafK-lfiEFGHIJ*, encodes the proteins involved in regulation and assembly of the basal body components and is analogous to the *flhAB-fliRQPNMEFGHIJ* genes in the *flag*-1 locus (Fig. 3) [7, 15]. The encoded orthologues among the 461 complete complement strains share 67.78% AAI. One Cluster 2 protein restricted to the *flag*-2 loci, LafK, has been predicted to serve as regulator of flagellum biosynthesis [15] and shares 67.23% AAI among the 461 strains with complete *flag*-2 loci. Cluster 2 also typically comprises fourteen genes, *lfgNMABCDEFGHIJKL*, which are orthologous to *flgNMABCDEFGHIJKL* in the *flag-*1 locus and encode flagellar structural proteins (Fig. 3) [12]. The *flag*-2 cluster 2 proteins show slightly greater variability than the cluster 1 genes, sharing 61.44% AAI, with four proteins, LfgN (chaperone), LfgM (Anti σ^28^ factor), LfgA (basal body P-ring protein) and LfgL (hook-associated protein) sharing < 50% AAI.

Cluster 3 comprises of the genes *lafWZABCDEFSTU*, which code for eleven proteins with substantially lower orthology (50.07% AAI) than those in Cluster 1 and 2. These include proteins involved in filament synthesis (LafABCD – orthologues of FliCDST), σ^28^ factor LafS (orthologue of FliA) and the motor proteins LafT and LafU (orthologues of MotA and MotB in the *flag*-1 locus) (Fig. 3). Also within this cluster are genes coding for the proteins LafW and LafZ, which represent a putative hook-associated protein and transmembrane regulator, respectively [15], orthologues of which are absent from the *flag*-1 locus. The latter proteins share lower AAI values of 44.57 and 38.89%, respectively.

### Gene and *en bloc* deletion may have resulted in non-functionality of the *flag-*2 system in some Enterobacterales taxa

While a substantial fraction of the *flag*-2 loci contain a complement of 39 conserved genes coding for proteins involved in flagellar biosynthesis and functioning, 22.13% of enterobacterial strains are missing at least one of these genes. For example, 22/67 *Y. enterocolitica* strains are missing the entire Cluster 1 (*lfhAB-lfiRQPNM-lafK-lfiEFGHIJ*), while 3/91 *Citrobacter* Clade A strains lack both Cluster 2 and Cluster 3. Transposition appears to be a major driver of the observed *en bloc* gene deletions. As such, twenty-five distinct transposase genes are localised within the Enterobacterales *flag*-2 loci. These belong to a range of different transposase families, including IS1, IS4, IS5, IS110 and Mu transposases and are integrated in diverse locations within the *flag*-2 loci. The reading frames of individuals genes could also be observed to be disrupted by transposase integration, with *lfgF* (20 strains) and *lfiG* (7 strains), being particularly prone.

Previous analyses showed that in many *Escherichia* and *Shigella* strains, a deletion has occurred within the reading frames of the *lfhA* and *lafU* genes which occur at the 5′ and 3′ ends of the *flag-*2 locus, respectively, resulting in loss of the remaining locus between the *lfhA* and *lafU* pseudogene fragments. The presence of direct repeats at the ends of this deletion suggest that this may have resulted through recombination events [15]. Blast analyses of the *lfhA* and *lafU* genes and proteins against the 4028 Enterobacterales strains showed that this occurs in the genomes of 531 (13.18%) of the strains (Fig. 1 – indicated by blue triangles). The *lfhA* and *lafU* pseudogenes are primarily found in those taxa where complete *flag-*2 loci are present. For example, of the 100 *E. coli* strains analysed, all 76 strains that lack *flag-*2 loci contain the truncated gene copies. Similarly, 50 (75.76%) of the 66 *Citrobacter* Clade A strains lacking *flag*-2 loci show evidence of its deletion. This suggests that the *flag-*2 locus is likely to have been a far more prevalent feature among the Enterobacterales (27.88%; 1123/4028 analysed strains) prior to *en bloc* deletion of the locus in a substantial number of strains. While large scale deletions are partially responsible for the difference in size and protein complement observed among the enterobacterial *flag*-2 loci, it can further be attributed to the integration of a substantial set of non-conserved cargo genes within the loci.

### The enterobacterial *flag*-2 loci are hotspots for integration of cargo genes

Alignment of the enterobacterial *flag*-2 loci and comparative analysis of their encoded protein complements revealed that, although extensive synteny and a substantial set of conserved proteins occur among these loci (Fig. 2), there are 349 distinct protein coding genes, which are not conserved among all enterobacterial *flag*-2 loci and which do not form part of the core set involved in flagellar biosynthesis and functioning. As such, they can be considered as cargo genes within the *flag*-2 loci. A substantial proportion (121 genes; 34.67% of cargo genes) of these genes code for hypothetical proteins and proteins containing domains of unknown function. However, BlastP searches against the NCBI non-redundant protein database and the Conserved Domain Database [19], identified proteins with a range of non-flagellar related functions within the *flag*-2 loci. For example, the *flag*-2 loci of twenty-one *Escherichia* strains incorporate genes coding for the restriction endonuclease EcoRII (pfam09019; E-value: 8.36E-98; Average size: 401 aa; AAI: 97.1%) and DNA cytosine methylase Dcm (PRK10458; E-value: 0.0; Average size: 474 aa; AAI: 98.6%). These function in cleaving DNA at a specific sequence and methylation of this sequence to prevent restriction and protect the bacterial cell from integration of bacteriophage and plasmid DNA [20]. Four *Pragia fontium* strains incorporate genes coding for the pilin protein FimA (PRK15303; E-value: 4.13E-03), periplasmic chaperone FimC (PRK09918; E-value: 3.07E-91) and usher protein FimD (PRK15304; E-value: 0.0).

Cargo genes are found interspersed throughout the *flag*-2 loci, usually in single or two gene clusters. However, two regions appear to be particularly prone to integration of cargo genes. The first variable region (VR1) occurs between the *flag*-2 gene clusters 1 and 2 (between *lfiJ* and *lfgN*), while the second (VR2) occurs at the 5′ end of cluster 3 (between *lafW* and *lafZ*) (Fig. 2). VR1 occurs in the *flag-2* loci of 382/592 (64.53%) enterobacterial strains and is particularly prevalent in members of the family *Budviciaceae* (7/7 strains), *Enterobacteriaceae* (310/316 strains) and *Hafniaceae* (28/28 strains), but are more restricted among the *flag*-2 loci of the *Erwiniaceae* (4/8 strains) and *Yersiniaceae* (33/225 strains; 14.67%). The VR1 regions vary in size between 0.7 and 18.9 kb (average size: 5.9 kb) and code for between one and twenty-three (average proteins: 5) proteins (Additional file 1: Table S3). This region shows evidence of having been derived through horizontal gene transfer, with G + C content deviations of − 15.1 to + 4.1% (average − 2.6%) and − 12.5 to + 4.2% (average − 3.6%) from the genomic and *flag-*2 G + C content, respectively. A total of 154 distinct proteins are encoded by the VR1 regions of the enterobacterial *flag*-2 loci. VR2 is more prevalent compared to VR1, occurring in 478/592 (80.74%) of all Enterobacterales. This includes the *flag*-2 loci of all *Budviciaceae* and *Hafniaceae* and 93.78, 72.47 and 37.5% of the *Yersiniaceae*, *Enterobacteriaceae* and *Erwiniaceae*, respectively. This variable region is typically smaller than VR1, ranging in size from 0.4 to 5.5 kb (average size: 0.6 kb) and coding for between one and six (average: 1 protein) distinct proteins. This region also shows evidence of horizontal acquisition, with an even more pronounced G + C deviation of − 13.6 to + 8.7% (average: − 4.7%) and − 15.4 to + 5.1% (average: − 4.7%) from the genomic and *flag*-2 locus G + C contents, respectively. VR2 codes for twenty-seven distinct proteins, with the majority of these (21/27; 77.78%) being hypothetical proteins or those containing domains of unknown function (Additional file 1: Table S3). By contrast, many of the proteins in VR1 share orthology with proteins involved in glycosylation and modification of the flagellar filament.

### The *flag*-2 variable region 1 (VR1) encodes the machinery for glycosylation, methylation and modification of the flagellum

The most prevalent genes among the (520/1826 total VR1 proteins; 28.48%) enterobacterial *flag*-2 VR1 regions are those that code for twenty-two distinct glycosyltransferases. Glycosyltransferase enzymes catalyze glycosidic bond synthesis resulting in the covalent attachment of a glycan to proteins or other sugars and are likely to contribute towards glycosylation of the main structural protein of the flagellum, flagellin [14, 15]. This posttranslational modification has been linked to a wide range of phenotypes, including surface recognition, adhesion, biofilm formation, antigen masking from immune response and virulence [14, 15]. Flagellin glycosylation is a relatively common feature among the *Enterobacteriaceae* and the *flag-*1 flagellar systems of 307/2000 enterobacteria (15.4%) were predicted to be glycosylated [14]. Here, 341/592 (57.60%) of the *flag*-2 loci contain genes coding for glycosyltransferases which are predicted to be involved in flagellin glycosylation. BlastP comparison against the CAZy database, using the dbCAN pipeline [21], classified these *flag*-2 proteins into five distinct glycosyltransferase families. Ten distinct proteins belong to the GT2 family (GT2–1 to GT2–10), members of which transfer a wide array of saccharides including mannose, galactose, N-acetylglucosamine, glucose and their derivatives [14, 22]. The most common of these GT2 glycosyltransferases is GT2–1, which occurs in 222 *Enterobacteriaceae* belonging to eight distinct genera. The VR1 of seventeen strains in six genera of *Enterobacteriaceae* and *Erwiniaceae* incorporate a gene coding for a glycosyltransferase of the GT4 family, which likewise transfer a broad range of sugars, including glucose, mannose and glucosamine [22]. A further 70 strains encode five distinct glycosyltransferase orthologues (GT9–1 to GT9–5) of the GT9 family, which incorporates lipopolysaccharide N-acetylglucosaminyltransferases and heptosyltransferases [22]. Another represented glycosyltransferase family, GT25 comprises four distinct orthologues (GT25–1 to GT25–4) in a total of twenty-six taxa and includes galactosyltransferases and proteins involved in lipopolysaccharide biosynthesis. Finally, fourteen strains incorporate two different types of GT32 (GT32–1 and GT32–2) family glycosyltransferases with a purported role in mannose, galactose and glucosamine saccharide transfer [22].

Given that distinct sugars can be incorporated by glycosyltransferases belonging to the different GT families, the type of sugars incorporated in the flagellin glycan cannot solely be determined on the basis of the type of glycosyltransferase present. Proteins that catalyse the synthesis of these sugar moieties may be localized in genomic locations other than the *flag*-2 locus. However, the VR1 region of 35 *Citrobacter* Clade A strains encodes orthologues of the sialyltransferase PM0188 (pfam11477; E-value: 2.54E-16) of *Pasteurella multocida*, suggesting that the flagellin glycan of the latter strains may incorporate neuraminic acid [23]. Orthologues of lipoteichoic acid synthase LtaS (LtaS1 and LtaS2; cd16015; 30 strains across three genera in the *Enterobacteriaceae* and *Hafniaceae*) and CDP-glycerol phosphotransferase TagB (TagB1 to TagB8; COG1887; 65 strains across thirteen genera in the *Enterobacteriaceae* and *Hafniaceae*) are encoded on the VR1 regions of *flag*-2 loci. Additionally this region encodes a glycerol-3-phosphate cytidylyltransferase TagD (cd02171; E-value: 4.09E-46) in 235 strains across ten genera and three families. These proteins are central to the synthesis of teichoic acid, phosphodiester-linked polyol glycopolymers that form a major part of the cell wall of most Gram-positive bacteria [24]. The presence of orthologues of genes involved in this function in VR1 suggest that these glycopolymers form part of the flagellin glycan of the enterobacterial *flag*-2 system.

While the exact glycan sugar moieties of the flagellin glycans are difficult to determine, the presence of orthologues of diverse proteins which may modify or substitute the glycan chains suggest the *flag*-2 flagellin glycan is heavily decorated as has been observed in the primary flagellar system of many enterobacteria as well as Gram-positive bacteria [14, 18]. Among the VR1-encoded proteins are orthologues of the acetyltransferases NeuD (Neuraminic acid acetyltransferase; TIGR03570; E-value: 2.65E-61; *Citrobacter* Clade A *werkmannii* AK-8), RimI (N-acetyltransferase; pfam00583; E-value: 1.69E-11; *Escherichia* sp. nov 1 E1642), OptS (*O*-phosphoseryl acetylase; PRK06253; E-value: 5.94E-03; two *Escherichia albertii* strains) and WbbJ (maltose *O*-acetyltransferase; cd04647; E-value: 3.33E-25; twelve *Citrobacter* Clade A strains), the pyruvyl transferase WcaK (pfam04230; E-value: 2.59E-16; fourteen *Citrobacter* Clade A strains) and transaminase WecE (dTDP-4-amino-4,6-dideoxygalactose transaminase; COG0399; E-value: 4.48E-103; twenty-nine *Enterobacteriaceae* and *Hafniaceae* strains). Furthermore, fifteen distinct methyltransferases belonging to the FkbM (FkbM1 to − 3; TIGR01444), Mtf11 (Mtf11–1-3; pfam08241), Mtf12 (pfam08242), Mtf23 (Mtf23–1 to − 4; pfam13489), Mtf24 (Mtf24–1 and − 2; pfam13578) and Mtf25 (Mtf25–1 and − 2; pfam13649) families are encoded in the *flag*-2 VR1 regions. This suggests that the *flag*-2 flagellin glycans are decorated with methyl, acetyl, amino and pyruvyl groups. An additional *N*-lysine methylase FliB has been observed to be relatively common among the *flag*-1 loci and flagellin methylation has been suggested to play a role in virulence in *Salmonella enterica* [14, 25]. Orthologous proteins, previously termed LafV (LafV1 to LafV10), occur in the *flag*-2 VR1 regions of 342/592 (57.58%) enterobacteria advocating that flagellin methylation is also a common phenomenon in the *flag*-2 flagellar system. While a range of functions have been ascribed to flagellin glycosylation and methylation for the primary flagellar system, its prevalence among the *flag*-2 loci suggests similar important roles in the latter system, but this needs to be confirmed experimentally.

### The enterobacterial *flag*-2 locus displays a complex evolutionary history of vertical transmission and horizontal acquisition

**Fig. 4:**
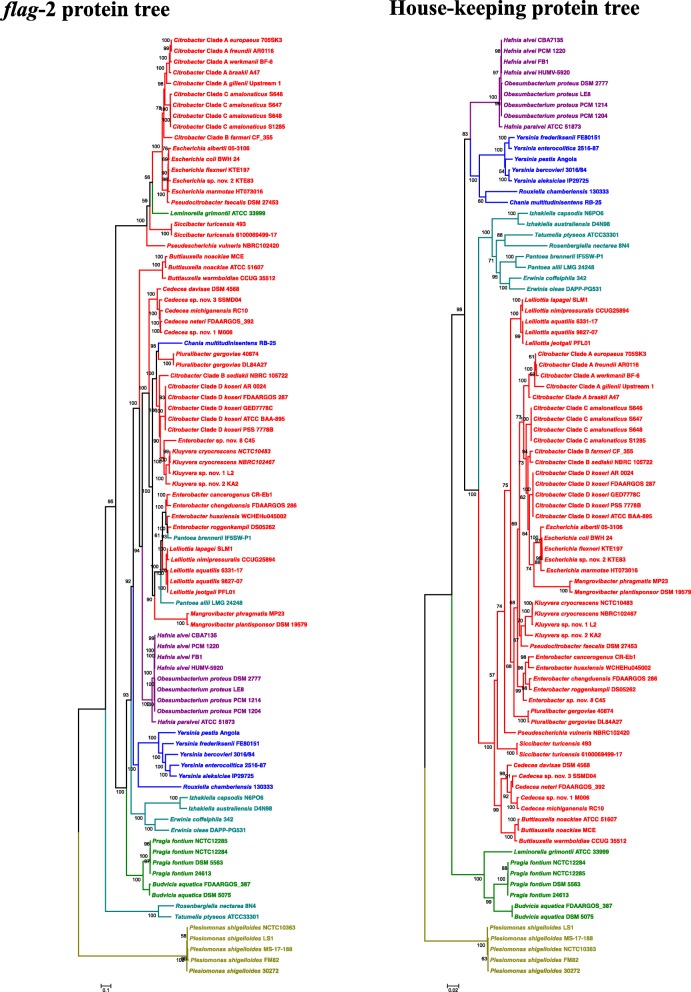
Comparison of conserved *flag*-2 protein tree and house-keeping protein tree. A ML phylogeny was constructed on the basis of a concatenated alignment of 32 conserved *flag-*2 proteins (left) as well as on the basis of the house-keeping proteins GyrB, InfB, RecA and RecB (InfB). The final alignments for the *flag*-2 protein tree and house-keeping proteins comprised of 5800 and 2893 amino acid sites, respectively. The best-fit evolutionary models LG + FI + G4 were used for construction of both phylogenies. Bootstrap values (*n* = 1000 replicates) are indicated. The strains and branches are coloured according to the family to which each belong

The relatively low prevalence of *flag*-2 loci among the Enterobacterales suggests that they may have been acquired through horizontal gene transfer (HGT) in a select set of taxa. Comparison of a phylogeny on the basis of 32 *flag*-2 proteins conserved among 87 taxa representative of six families and 27 genera and a house-keeping protein phylogeny shows that there is congruence at the genus and family level for some taxa (Fig. 4). For example, *Yersinia* and *Rouxiella* (*Yersiniaceae*), *Hafnia* and *Obesumbacterium* (*Hafniaceae*) and *Pragia* and *Budvicia* (*Budviciaceae*) form cohesive clades in both phylogenies (Fig. 4). This suggests the *flag*-2 loci have been maintained via vertical transmission through speciation events in these evolutionary lineages. However, a more complex evolutionary history can be ascribed to the *flag*-2 loci of some taxa. The *flag*-2 locus of *Leminorella grimontii* ATCC 33999 (*Budviciaceae*) clusters with those of the *Enterobacteriaceae Citrobacter* Clade A and C and *Escherichia*, while that of *Chania multitudinisentens* RB-25 clusters with *Pluralibacter* (*Enterobacteriaceae*). Similarly, the *flag*-2 loci of *Pantoea brennerii* IF5SW-P1 and *Pantoea allii* LMG 24248 cluster distinctly with *Enterobacter* and *Lelliottia* spp. and not with the other taxa, *Erwinia* and *Izhakiella*, of the *Erwiniaceae*. Moreover, the *Enterobacteriaceae* form three distinct clades when considering their *flag-*2 loci (Fig. 4). This suggests that *flag*-2 loci have been derived through HGT events in some of these taxa. Genomic regions derived through recent HGT events are often typified by G + C contents that vary substantially from the rest of the genome [26]. In general, the *flag-*2 loci of all 592 enterobacterial strains have a G + C content only marginally above (average G + C deviation = 0.5%; range: − 4.0 to + 5.0%) that of the remainder of the genome (Additional file 1: Table S2). This may be attributed in part to the distinct G + C content of the VR1 and VR2 regions. Exclusion of these regions, however, resulted in even more pronounced G + C deviations for the *flag*-2 loci (average G + C deviation: + 1.1%; range: − 4.0 to + 5.8%) suggesting horizontal acquisition in a more substantive set of taxa. This is particularly evident in the *Enterobacteriaceae*, where the *flag*-2 loci have an average G + C content 2.1% (range − 3.1 to + 5.8%) above that of the rest of the genome. It is furthermore evident in those taxa placed in distinct family clades in the *flag*-2 tree, including *L. grimontii* ATCC 33999 (G + C deviation: + 2.5%), *C. multitudinisentens* RB-25 (G + C deviation: + 2.4%) and *P. allii* LMG 24248 (G + C deviation: − 3.6%). In one of the *Enterobacteriaceae* clades in the *flag*-2 tree, G + C deviations are pronounced in *Escherichia* (G + C deviation: + 4.8%), *Pseudocitrobacter* (G + C deviation: + 2.8%) and *Siccibacter* (− 2.9%). By contrast, the *flag-*2 loci in taxa belonging to Citrobacter Clade A have average G + C deviations of 0.0%, suggesting that the latter may represent ancestral *flag*-2 loci which have been derived through HGT in the former strains. However, among the *flag-*2 loci of *Escherichia* spp. (124 strains) and *Yersinia* spp. (223 strains) G + C deviations of + 2.6 to + 5.8% and − 3.0 to + 1.0% could be observed, indicating complex evolutionary histories for the *flag*-2 loci even within these apparently stable lineages.

### The *flag*-2 flagellar system may represent a multi-functional machine among the Enterobacterales

A broad range of functions have been ascribed to the *flag*-1 flagellar system, including swimming and swarming motility, adhesion, biofilm formation, host invasion and colonization [11]. By contrast, little is known about the function(s) of the secondary flagellar system among the Enterobacterales. The deletion (between *lfhA* and *lafU*) in *Escherichia* or frameshift mutation in *Escherichia* sp. nov. 2 strain 042 was deemed to inactivate the *flag*-2 system, rendering it non-functional [15]. Phage integration within both the *flag*-1 and *flag*-2 loci of *C. rodentium* has been suggested to have resulted in their non-motile phenotype [27]. This is in line with the concept that synthesis and rotation of the primary (*flag-*1) system, represents a significant metabolic burden on the bacterial cell, accounting for 2.1% of the overall energy requirement in *E. coli*, where the *flag*-2 system may not provide a selective advantage to the cell [15, 28]. However, several lines of evidence suggest the *flag*-2 system plays several important roles in other enterobacterial taxa. The *flag*-2 flagella of *Plesiomonas shigelloides* have been shown to be essential for swarming motility [29]. Here we have observed *flag*-2 loci in the genomes of members of the family *Budviciaceae* (two strains of *Budvicia aquatica* and four strains of *P. fontium*) and *Erwiniaceae* (*Tatumella pytseos* ATCC 33301), which lack the *flag*-1 flagellar system but have nevertheless been described as being capable of flagellar motility [2]. By contrast, *L. grimontii* ATCC 33999 (*Budviciaceae*) and *Rouxiella chamberiensis* 13,033 (*Enterobacteriaceae*), which lack a *flag*-1 locus but retain a *flag*-2 locus, have been described as non-motile [2]. The *flag*-2 locus of the latter strain is, however, missing the *lafZ* gene.

While the *flag*-2 loci of many of the enterobacterial taxa contain deletions or genes with disrupted reading frames, a substantial number (77.87% of strains containing *flag*-2 loci) appear to encode the full complement of proteins for the synthesis and functioning of this flagellar system. Many of these have functional *flag*-1 loci, and as such the *flag*-2 system may play roles other than in motility in these taxa. The *flag*-1 system has been implicated in the secretion of virulence factors by pathogenic bacteria, including the phospholipase YplA in *Y. enterocolitica*, the lipase XlpA and antibacterial xenocin in the entomopathogen *Xenorhabdus* and invasion antigen (Cia) protein in *Campylobacter* spp. [4, 30, 31]. Analysis of the proteins encoded in the VR1 and VR2 regions of the *flag*-2 loci revealed a number of putative secretion targets among the cargo proteins. The VR1 region of the *Escherichia fergusonii* YH17130 *flag*-2 locus incorporates a gene coding for a Type VI secretion system (T6SS) effector protein VgrG (COG3501; E-value: 0.0). This gene is flanked by genes coding for a 1556 amino acid Rhs-domain containing protein (COG3209; E-value: 8.53E-44) and polymorphic toxin immunity protein Imm26 (pfam15428; E-value: 1.7E-15). Rhs-domain proteins represent toxins that have been found to be involved in inter-bacterial competition and are also secreted via the T6SS, where the Imm26 protein may serve as a protective mechanism against autotoxicity [32]. Furthermore, the VR1 and VR2 regions in the *flag*-2 locus of two and three *Lelliottia* spp., respectively encode orthologues of two distinct Haemolysin co-regulated (Hcp) proteins (Hcp1; pfam05638; E-value: 1.48E-30; Hcp2; COG3157; E-value: 1E-40), which likewise serve as secretion effector proteins [33]. The presence of distinct secreted effector proteins in a number of strains imply a putative role for the *flag*-2 system in secretion. Furthermore, within the VR1 regions of the *Escherichia* sp. nov. 1 strain E1642 and *Lelliottia aquatilis* 6331–17 are genes coding for an orthologue of pesticin (Pst; CD16903; E-value: 1.88E-73), a phage lysozyme-like bacteriocin from *Y, pestis* and an orthologue of the RNase toxin Ntox44 (pfam15607; E-value: 4.52e-16) [34, 35], respectively, ascribing a role in antibacterial activity or inter-bacterial competition to the *flag*-2 system. Finally, four *P. fontium* (*Budviciaceae*) incorporate a gene coding for an 879 aa orthologue (96.6% AAI) of the autotransporter MisL (PRK15313; E-value: 6.51E-97). In *S. enterica* serovar Typhimurium this protein plays a role in adherence, aggregation and biofilm formation [36].

## Discussion

The *flag-*2 locus is a relatively prevalent phenomenon among the Enterobacterales, comprising of a syntenous locus coding for 39 well-conserved proteins. Comparative genomic analyses revealed that frequent transposon integration has occurred within single genes in the locus, as well as *en bloc* deletion between the partial reading frames of *lfhA* and *lafU*. The *en bloc* deletions and gene disruptions can be envisaged to have a detrimental effect on functionality of the *flag*-2 system, as has been observed in *Escherichia* sp. nov 2 strain 042 [15]. Furthermore, pervasive deletion of the locus suggests that it may have been a far more common genomic feature and hints at a complex evolutionary history for the locus. A recent study has identified orthologous *flag*-2 loci among members of the alpha-proteobacterial order Rhizobiales and furthermore, they are similar to the lateral flagellar system of *Vibrio* and *Aeromonas* spp. [15, 37]. A more comprehensive analysis across a broader taxonomic scope may shed further light on the evolution of the *flag*-2 system.

Phylogenetic analysis on the basis of the conserved proteins encoded on the *flag*-2 loci suggest disparate evolutionary histories for these loci among the enterobacterial taxa, with the *flag*-2 loci some taxa likely being derived through HGT events, while some lineages shows evidence of retention of this locus through vertical transmission. Analysis of the *flag*-1 protein complement of forty-one motile species across eleven bacterial phyla showed extensive sequence similarity between the twenty-four core proteins conserved among all taxa [38]. It has thus been postulated that a few genes, or even a single precursor, may have given rise to the full complement of genes required for the synthesis of the primary flagellar system through gene duplications, gene fusions and the recruitment of novel genes [38, 39]. It is plausible that similar evolutionary processes may have given rise to the extant *flag*-2 loci, which may explain the complex evolutionary histories of these loci among the Enterobacterales.

A communal feature of the *flag*-2 loci of most enterobacterial taxa is the presence of cargo genes, integrated mainly in two variable regions, VR1 and VR2, which mainly encode the machinery for flagellin glycosylation, which as observed in the *flag*-1 locus, likely represents a common posttranslational feature of this flagellar system. A wide range of functions have been ascribed to this feature in the primary flagellar system [14], but its role in the secondary flagellar system remains to be elucidated. Also among the cargo genes are those which provide hints on the potential functions of the secondary flagellar system. While earlier evidence largely advocated for the non-functionality of the *flag*-2 system, the elucidation of a functional *flag*-2 system in *P. shigelloides* [29] and the presence of *flag*-2 loci containing the full complement of conserved flagellar biosynthesis and operational genes suggest this may represent a multi-functional system with several potential roles. As many of the taxa with intact *flag*-2 loci represent both clinical and plant pathogens, knock-out mutagenesis and further characterization are imperative for a deeper understanding of this intriguing flagellar system.

## Conclusions

Highly conserved and syntenous secondary flagellar (*flag*-2) loci occur in ~ 15% all screened members of the order Enterobacterales and likely represents important functional features in the taxa that incorporate them. Variable regions within the *flag*-2 loci code for proteins with roles in posttranslational modification of the flagellar system as well as those that provide hints into the functions of the *flag*-2 system. Future work will focus on determining the biological role(s) of the secondary flagellar system in members of the order Enterobacterales.

## Methods

### Identification of *flag*-2 loci among the Enterobacterales

The genomes of 4028 members of the order Enterobacterales were selected for analysis. Selection was based on the current genomic taxonomy in the Genome Taxonomy DataBase (GTDB Release 04-RS89) [40] and provided coverage of all eight families (75 genera) and *Plesiomonas* (family unassigned). Up to 100 strains representative of each species were included in the analysis. The presence of *flag*-2 loci was determined by local tBlastN analyses with the LafK (CAH19120.1), LafW (CAH19144.1), LafZ (CAH19147.1) LafA (CAH19148.1) and LafB (CAH19149.1) proteins of *E. coli* 042 [15] using BioEdit v.7.2.5 [41]. The *lafK*, *lafW* and *lafZ* genes are conserved among most strains that harbour *flag-*2 loci and serve as distinguishing factors, as orthologous genes are absent from the *flag-*1 locus [15]. For those genomes with Blast hits for the above proteins, the complete *flag*-2 loci were identified by searching the annotated Genbank genome sequences up- and downstream of these genes. These complete *flag*-2 loci were structural annotated using the Prokaryotic GeneMark.hmm v.2 server [42] and the G + C% contents of each of the loci were calculated using BioEdit v.7.2.5 [41].

### Comparative genomic analyses of the enterobacterial *flag-*2 loci

Orthologues in the *flag-*2 loci protein datasets of twenty-five strains were identified using Orthofinder v 1.1.4 [43]. The distinct orthogroups were functionally annotated by BlastP analyses against the NCBI non-redundant (nr) protein database and the NCBI Conserved Domain Database (CDD) using Batch CD-Search [19]. Furthermore, proteins with predicted functions in carbohydrate catabolism were compared against the Carbohydrate Active Enzymes (CAZy) database using the dbCAN server [21] to classify putative glycosyltransferases and glycosidases.

### Phylogenetic analyses

A maximum likelihood (ML) phylogeny of the 4028 enterobacterial strains incorporated in this study was constructed using the concatenated amino acid sequences of four conserved house-keeping proteins, namely Gyrase B (GyrB), translation initiation factor IF-2 (InfB), recombinase A (RecA) and RNA polymerase beta subunit (RpoB). The presence/absence/deletion of the *flag*-2 locus was mapped onto this phylogeny. Similarly a ML phylogeny was constructed on the basis of 32 proteins conserved among 87 enterobacterial taxa representative of the taxonomic spectrum of the order, with up to five strains or species per genus included in the analysis. Individual proteins were aligned using the M-Coffee implementation of T-Coffee [44]. The aligned proteins were concatenated and poorly aligned blocks were removed using Gblocks v. 0.91b [45]. The curated concatenated alignments were then used to construct ML phylogenies using IQTree v. 1.6.11 [46] using the appropriate evolutionary model predicted using ModelFinder [47] and bootstrap support using UFBoot2 (*n* = 1000 replicates) [48].

## Supplementary information

**Additional file 1: Table S1.** Presence/absence of *flag*-2 loci among 4028 strains belonging to eight families and 72 genera. The presence of *flag*-1 loci is also indicated. The previous taxonomy denotes the taxonomy according to the NCBI genome database, while the current taxonomy is according to the Genome Taxonomy Database (GTDB). The isolation source as well as habitat/lifestyles of the different strains are given. **Table S2.** Molecular characteristics of the *flag*-2 loci among 592 taxa in the Enterobacterales. The sizes of the *flag*-2 loci, variable regions VR1 and VR2, their G + C contents (%) and G + C deviation (%) from the genome are shown. The number of predicted proteins encoded on each of these *flag*-2 fractions are also shown. **Table S3.** Characteristics of the cargo genes encoded in the variable regions VR1 and VR2 and elsewhere in the enterobacterial *flag*-2 loci. The number of strains and the families/genera in which each protein occurs within the *flag*-2 loci are indicated, as well as the average amino acid identities among enterobacterial orthologues. Conserved domains present in each cargo protein as determined by BlastP analysis against the Conserved Domain Database are shown.

## Data Availability

All genome sequences incorporated in this study are publically available in the NCBI Genome database. The NCBI accession numbers for the contigs/chromosomes on which the target loci are found are indicated in Additional file 1: Table S1.

## Abbreviations

AAI: Amino Acid Identity
GT: Glycosyltransferase
HGT: Horizontal Gene Transfer
ML: Maximum Likelihood
Mtf: Methyltransferase
